# Molecular mechanism of cytotoxicity induced by Hsp90-targeted Antp-TPR hybrid peptide in glioblastoma cells

**DOI:** 10.1186/1476-4598-11-59

**Published:** 2012-08-22

**Authors:** Tomohisa Horibe, Aya Torisawa, Masayuki Kohno, Koji Kawakami

**Affiliations:** 1 Department of Pharmacoepidemiology, Graduate School of Medicine and Public Health, Kyoto University, Kyoto, Japan

**Keywords:** Client proteins, Heat shock protein 90, Hybrid peptide, Molecular chaperone, Unfolded protein response, Glioblastoma

## Abstract

**Background:**

Heat-shock protein 90 (Hsp90) is vital to cell survival under conditions of stress, and binds client proteins to assist in protein stabilization, translocation of polypeptides across cell membranes, and recovery of proteins from aggregates. Therefore, Hsp90 has emerged as an important target for the treatment of cancer. We previously reported that novel Antp-TPR hybrid peptide, which can inhibit the interaction of Hsp90 with the TPR2A domain of Hop, induces selective cytotoxic activity to discriminate between normal and cancer cells both *in vitro* and *in vivo*.

**Results:**

In this study, we investigated the functional cancer-cell killing mechanism of Antp-TPR hybrid peptide in glioblastoma (GB) cell lines. It was demonstrated that Antp-TPR peptide induced effective cytotoxic activity in GB cells through the loss of Hsp90 client proteins such as p53, Akt, CDK4, and cRaf. Antp-TPR also did not induce the up-regulation of Hsp70 and Hsp90 proteins, although a small-molecule inhibitor of Hsp90, 17-AAG, induced the up-regulation of these proteins. It was also found that Antp-TPR peptide increased the endoplasmic reticulum unfolded protein response, and the cytotoxic activity of this hybrid peptide to GB cells in the endoplasmic reticulum stress condition.

**Conclusion:**

These results show that targeting of Hsp90 by Antp-TPR could be an attractive approach to selective cancer-cell killing because no other Hsp90-targeted compounds show selective cytotoxic activity. Antp-TPR might provide potent and selective therapeutic options for the treatment of cancer.

## Background

Malignant gliomas are the most commonly diagnosed malignant adult primary brain tumors, and median survival for glioblastoma (GB) is 12–15 months [1]. Targeted therapies, as single agents, have failed to offer long-term survival benefit, despite objective initial responses [2]. Heterogeneity and a complex molecular pathology of GB contribute to the lack of therapeutic success. It was previously reported that GB cells were dependent on a range of activated oncoproteins and signaling pathways which require heat-shock protein 90 (Hsp90) function [3].

Hsp90 is an abundant cytosolic molecular chaperone found within multimeric chaperone complexes known to participate in regulating protein homeostasis in cells. It is also well known that Hsp90 assists maturation of more than 200 proteins, which include transmembrane tyrosine kinase (Her2 and EGFR), metastable signaling proteins (Akt, K-ras, and Raf-1), mutated signaling proteins (p53 and v-Src), chimeric signaling proteins (Bcr-Abl), cell-cycle regulators (Cdk4 and Cdk6), and steroid receptors (androgen, estrogen, and progesterone receptors) [4-8]. Therefore, Hsp90 plays a unique role in cellular homeostasis, and consequently Hsp90 has emerged as a promising anticancer target.

On the other hand, it is also well known that cells respond to a wide variety of stresses through the transcriptional activation of genes that harbor stress elements in their promoters, and cells can also respond to stresses that are specific to individual organelles. For example, the accumulation of misfolded or unfolded proteins in the endoplasmic reticulum (ER) activate the ER unfolded protein response (erUPR) [9,10]. The erUPR is a complex signaling network that enhances cell survival by limiting the accumulation of unfolded or misfolded proteins in the ER [9,10]. The erUPR has three signaling pathways – inositol-requiring 1 (IRE1), PKR-like ER kinase (PERK), and ER-localized transmembrane protein ATF6 [9,10] – wherein PERK plays a major role in ER stress-induced translational attenuation [11]. ATF6 is activated by proteolysis and binds in the presence of NF-Y directly to a *cis*-acting element (CCAAT-N9-CCACG) to induce ER stress-inducible proteins which include molecular chaperones [12], whereas IRE1 mediates the unconventional splicing of XBP1 mRNA, thereby converting it to a potent erUPR transcriptional activator [13]. These transcription factors lead to coordinated induction of diverse erUPR target genes, such as the ER-resident molecular chaperones glucose-regulated proteins 78 (GRP78; also known as Bip) and 94 (GRP94), for cell survival [14,15]. However, the erUPR also induces the up-regulation of the *chop* gene, encoding a bZIP transcriptional factor CHOP (C/EBP homology protein), which is regulated by a number of transcriptional and translational mechanisms [16]. The induction of CHOP by the erUPR can lead to the transcriptional activation of Bim, leading in turn to apoptosis in the case of intolerable levels of the erUPR in the cells [17]. A broad range of cancer-types rely on ER protein-folding machinery to correctly fold key signaling pathway proteins, and erUPRs are strongly induced in various tumors [18]. Recently, accumulating evidence has demonstrated that the erUPR is an important mechanism required for cancer cells to maintain malignancy and therapy resistance. Hence, the erUPR may be also a significant target by which to improve cancer chemotherapy [19].

We previously reported that a newly designed Antp-TPR hybrid peptide inhibits the interaction of Hsp90 with tetratricopeptide repeat 2A domain (TPR2A) of p60/Hsp-organizing protein (Hop), has selective cytotoxic activity that allows it to discriminate between cancer and normal cell lines, and induces effective antitumor activity in a xenograft model of human pancreatic cancer in mice [20]. However, the detailed mechanism of cancer-cell-killing by Antp-TPR peptide still remains obscure. Recently it was reported that the Hsp90 antagonist geldanamycin and its derivative 17-allylamino-demethoxygeldanamycin (17-AAG) lead to ER stress-induced apoptosis in rat histiocytoma [21], whereas it was also reported that retaspimycin (IPI-504), which is a novel and soluble type of Hsp90 inhibitor derived from geldanamycin, blocks the UPR in multiple myeloma cells [22]. It is important for the further elucidation of cancer treatment targeting Hsp90 to address the functional mechanism of cancer-cell killing by Antp-TPR hybrid peptide. Here we report the mechanisms that Antp-TPR hybrid peptide uses to induce cancer-cell killing through the loss of Hsp90 client proteins such as p53, Akt, CDK4, and cRaf on GB cells. We also show that Antp-TPR hybrid peptide increases the erUPR and cytotoxic activity towards GB cells in the erUPR condition.

## Results

### Cytotoxic activity of Antp-TPR hybrid peptide to GB cell lines

**Figure 1:**
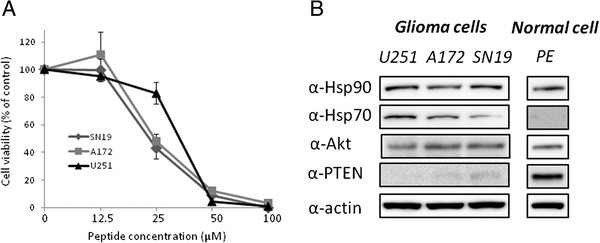
**Cytotoxic activity of Antp-TPR hybrid peptide to GB cells.** (**A**) Viability of GB cells (U251, A172, and SN19) treated with Antp-TPR peptide. Cells were incubated with Antp-TPR peptide at the indicated concentrations, and analyzed for cell viability as described in the Materials and Methods section. Data represent the mean ± SD from experiments performed in triplicate. (**B**) Analysis of Hsp90, Hsp70, PTEN, and Akt expression in GB and normal cell lines. Cell extracts from the indicated GB and normal PE (ACBRI 515) cell lines were examined for Hsp90, Hsp70, PTEN, and Akt expression by Western-blot analysis with corresponding antibodies. β-Actin was used as the loading control. Bands were visualized by chemiluminescence as described in the Materials and Methods section.

First we confirmed the previous report by Ohgaki [23] that the phosphoinositide 3-kinase (PI3K) pathway is often constitutively active in GB as a result of mutations and loss of a tumor suppressor, PTEN, compared with the other cancer cell lines (Additional file 1). Because of this phenomenon, it is assumed that GB is one of the most malignant cancers with a very poor prognosis. We then examined the cytotoxic activity and mechanism of Antp-TPR peptide in GB cells. As shown in Figure 1A, addition of Antp-TPR peptide to GB cells (U251, A172, and SN19) resulted in concentration-dependent cytotoxicity, and at 50 μM all the cells tested lost their viability. The IC_50_ values of Antp-TPR peptide in the GB cell lines, U251, A172, and SN19 were 26–36 μM, as shown in Table 1. When we examined the expression levels of Hsp70, Hsp90, and Akt, which is one of the client proteins of Hsp90 in GB cell lines, it was found that the endogenous expression levels of these proteins were equally unremarkable, and that PTEN was lost in these GB cells compared with the other cancer and normal cell lines (Figure 1B and Additional file 1).

**Table 1:** **Inhibitory concentration (IC**_**50**_**) of Antp-TPR peptide of glioma cells **

**Cell line**	**IC**_**50**_ (μM)^*^
SN19	26.53 ± 5.78
A172	28.27 ± 7.23
U251	35.70 ± 0.92

^*^Results are the mean of three independent experiments and each performed in triplicate.

### Cytotoxic mechanism of Antp-TPR in GB cells

**Figure 2:**
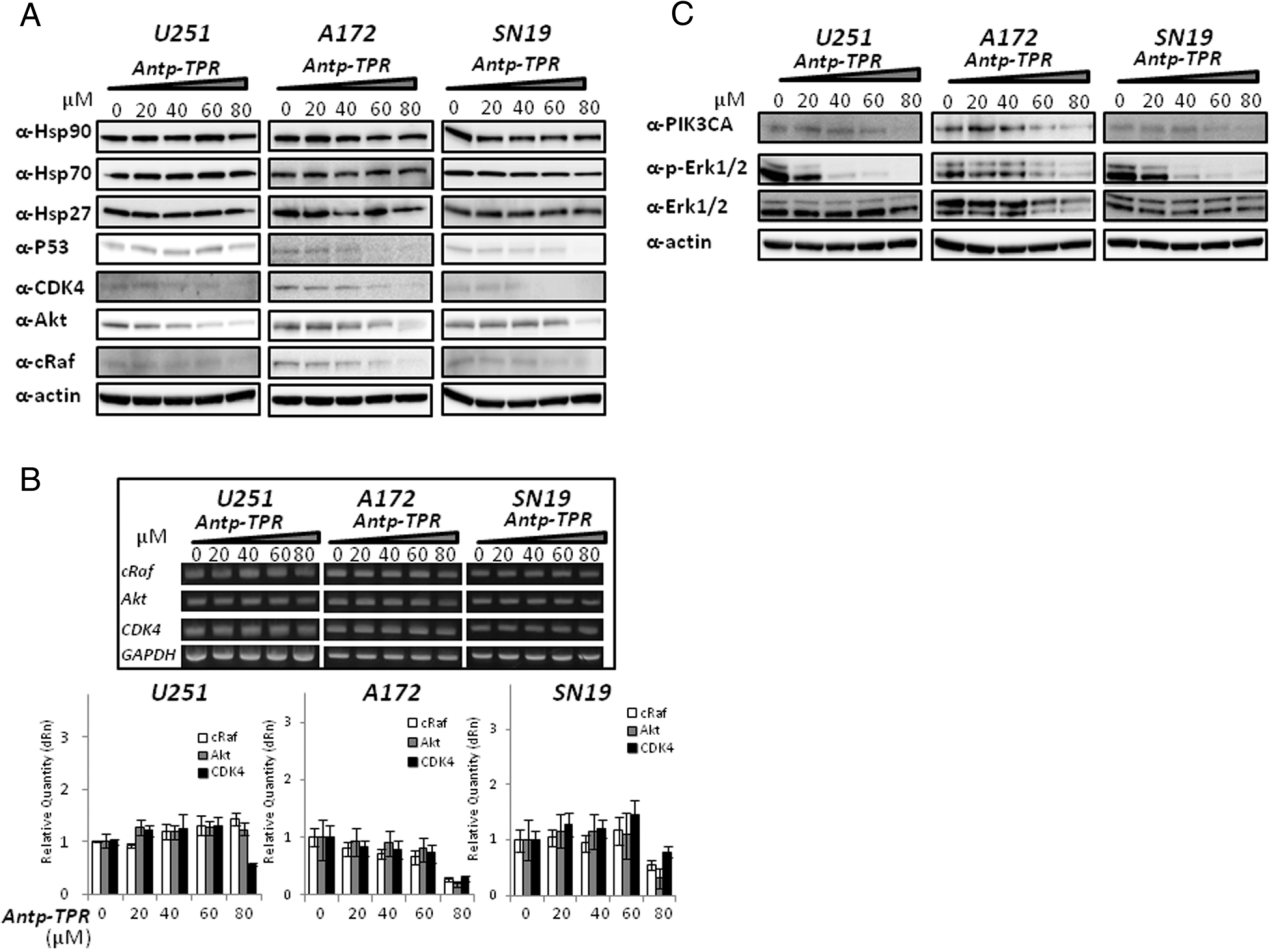
**Loss of Hsp90 client proteins.** (**A**) GB cells (U251, A172, and SN19) were treated with or without Antp-TPR peptide at the indicated concentrations, and examined by Western-blot analysis for expression of Hsp90, Hsp70, Hsp27, p53, CDK4, Akt, cRaf, and β-actin using corresponding antibodies. (**B**) Effect of Antp-TPR peptide on the transcriptional level of Hsp90 client proteins. Total RNA isolated from GB cells treated with or without Antp-TPR peptide at the indicated concentrations were reverse transcribed to cDNA, and then assessed by PCR (inset) or real-time quantitative PCR using specific primers as described in the Materials and Methods section. GAPDH served as an internal control. (**C**) Effect of Antp-TPR peptide on the Erk1/2 pathway. GB cells were treated with or without Antp-TPR peptide as indicated, and examined by Western-blot analysis for expression of PI3K, Erk1/2, or phosphorylated (p-) Erk1/2 using corresponding antibodies. β-Actin was used as the loading control. All bands of the Western-blot analysis were visualized by chemiluminescence.

We previously demonstrated that Antp-TPR peptide induced cancer-cell death through the loss of client proteins of Hsp90 such as CDK4 and Akt in breast cancer T47D cells [20]. These results prompted us to investigate the detailed mechanism of GB cell death as caused by this peptide. As shown in Figure 2A, treatment of GB cells (U251, A172, and SN19) with Antp-TPR resulted in a decrease in the expression of Hsp90 client proteins including p53, CDK4, Akt, and cRaf, in a concentration-dependent manner as compared with control untreated cells. Whereas Antp-TPR did not affect the expression levels of Hsp90, Hsp70, and Hsp27 proteins in GB cells after treatment with this peptide, 17-AAG did induce the up-regulation of these heat-shock proteins (Additional file 2). We next examined the effect of Antp-TPR on transcriptional level of Hsp90 client proteins (cRaf, Akt, and CDK4). Interestingly, it was found that the Antp-TPR peptide almost did not affect the transcription levels of these proteins until it reached 60 μM, and decreased the levels of mRNA for these proteins except for the levels of cRaf and Akt in U251 cells at higher concentrations (80 μM) (Figure 2B). These results indicate that Antp-TPR induces the loss of Hsp90 client proteins through the degradation of both protein and mRNA levels in GB cells. In addition, it was also found that the treatment of GB cells with Antp-TPR did not activate the Erk pathway, which is important for the differentiation and proliferation of cancer cells (Figure 2C).

### Molecular diversity of the cancer-cell-killing mechanism of Antp-TPR peptide and 17-AAG

**Figure 3:**
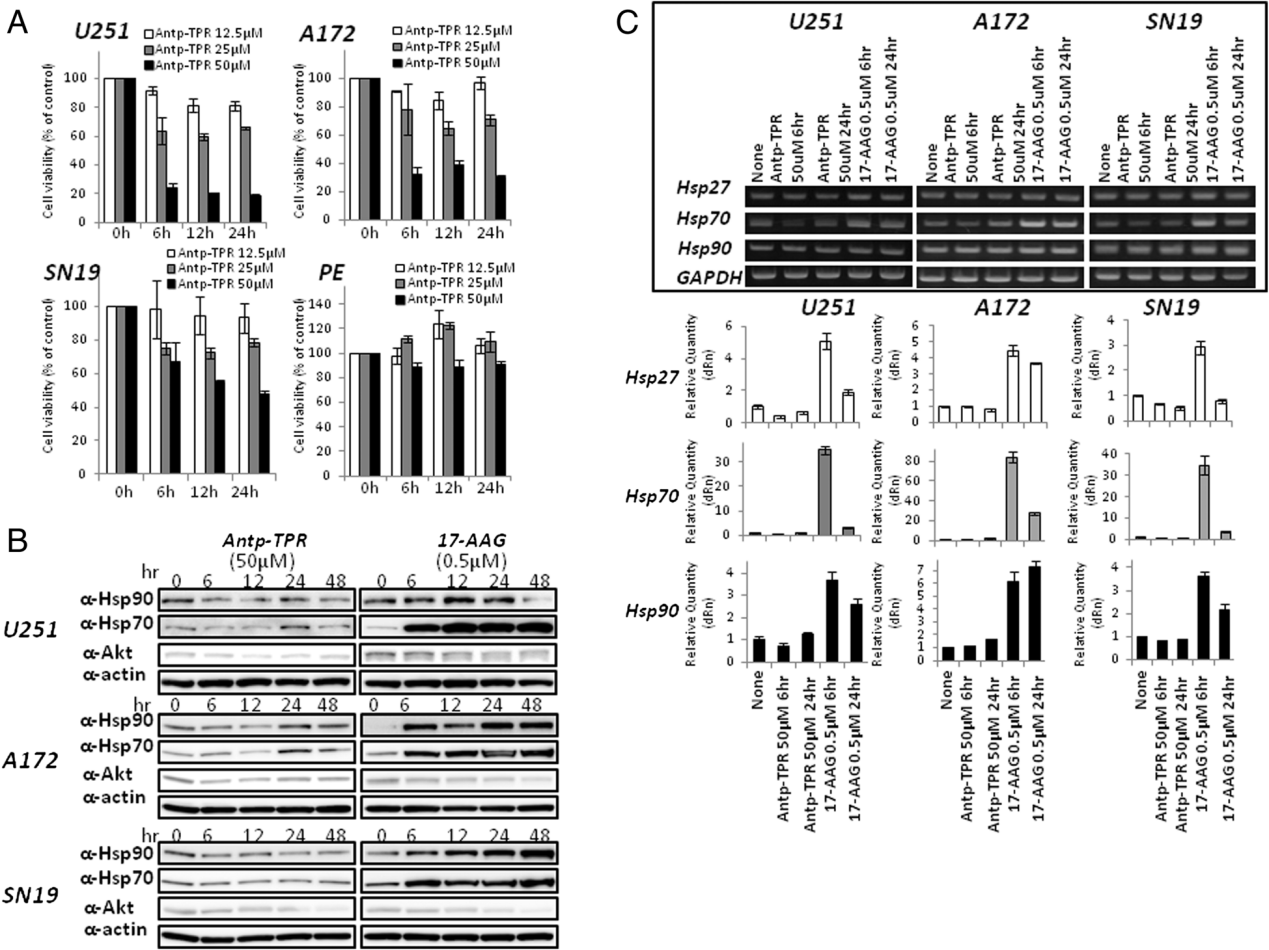
**Molecular diversity of the cancer-cell-killing mechanism of Antp-TPR peptide and 17-AAG.** (**A**) Time course of cell viability after the treatment with Antp-TPR peptide. GB (U251, A172, and SN19) and normal PE (ACBRI 515) cells were treated with Antp-TPR at the indicated concentrations, and then analyzed for cell viability by WST-8 assay. Data represent the mean ± SD from experiments performed in triplicate. (**B**) Induction of Hsp70 and Hsp90 by 17-AAG. GB cells were incubated with or without Antp-TPR peptide or 17-AAG for the indicated times, and extracts were examined by Western-blot analysis for expression of Hsp90, Hsp70, and Akt with their corresponding antibodies. β-Actin was used as the loading control. Bands were visualized by chemiluminescence. (**C**) Effect of Antp-TPR peptide and 17-AAG on transcription levels of Hsp27, Hsp70, and Hsp90 proteins. Total RNA isolated from GB cells treated with or without Antp-TPR peptide or 17-AAG at the indicated concentrations and time were reverse transcribed to cDNA, and then assessed by PCR (inset) or real-time quantitative PCR using specific primers as described in the Materials and Methods section. GAPDH served as an internal control.

The rate of Antp-TPR hybrid peptide-mediated cancer-cell killing was further investigated. A 6 h exposure of U251 and A172 cells to Antp-TPR (50 μM) was sufficient to induce high levels of cytotoxic activity to kill cells (approximately 70-80% decrease in cell viability) (Figure 3A). And, the decrease of cell viability with time dependent manner was not found in normal PE cells after the treatment with Antp-TPR (Figure 3A). In contrast, treatment with 17-AAG did not substantially affect cell viability, even after 24 h of treatment; however, a 48 h exposure of GB cells to 17-AAG resulted in an approximately 40–50% decrease in cell viability (Additional file 3A). It is known that the treatment of cancer cells with 17-AAG induces the up-regulation of Hsp70, and it is suggested that compensatory up-regulation of this protein is likely to correlate with the decrease of anticancer activity [24,25]. As shown in Figure 3B, 0.5 μM of 17-AAG quickly induced the up-regulation of Hsp70 within 6 h of treating U251, A172, and SN19 cells with this compound, whereas Antp-TPR did not cause the up-regulation of Hsp70 and Hsp90 proteins (Figure 3B). The up-regulation of these heat-shock proteins (Hsp90, Hsp70, and Hsp27) in GB cells by 17-AAG was also confirmed in transcriptional levels of mRNA, and Antp-TPR did not almost affect the transcriptional levels of these proteins (Figure 3C). In addition, the cytotoxic activity of Antp-TPR to U251 cells was not affected in the presence of PES, which was identified recently as a Hsp70 inhibitor by Leu et al. [26,27], although the cytotoxic activity of 17-AAG was increased in this condition (Additional file 3B). These results suggest that the Antp-TPR hybrid peptide has a different cancer-cell-killing mechanism than 17-AAG, and so might have an additional advantage compared with other Hsp90-targeted small compounds.

### Increase of the erUPR by Antp-TPR peptide in GB cells

**Figure 4:**
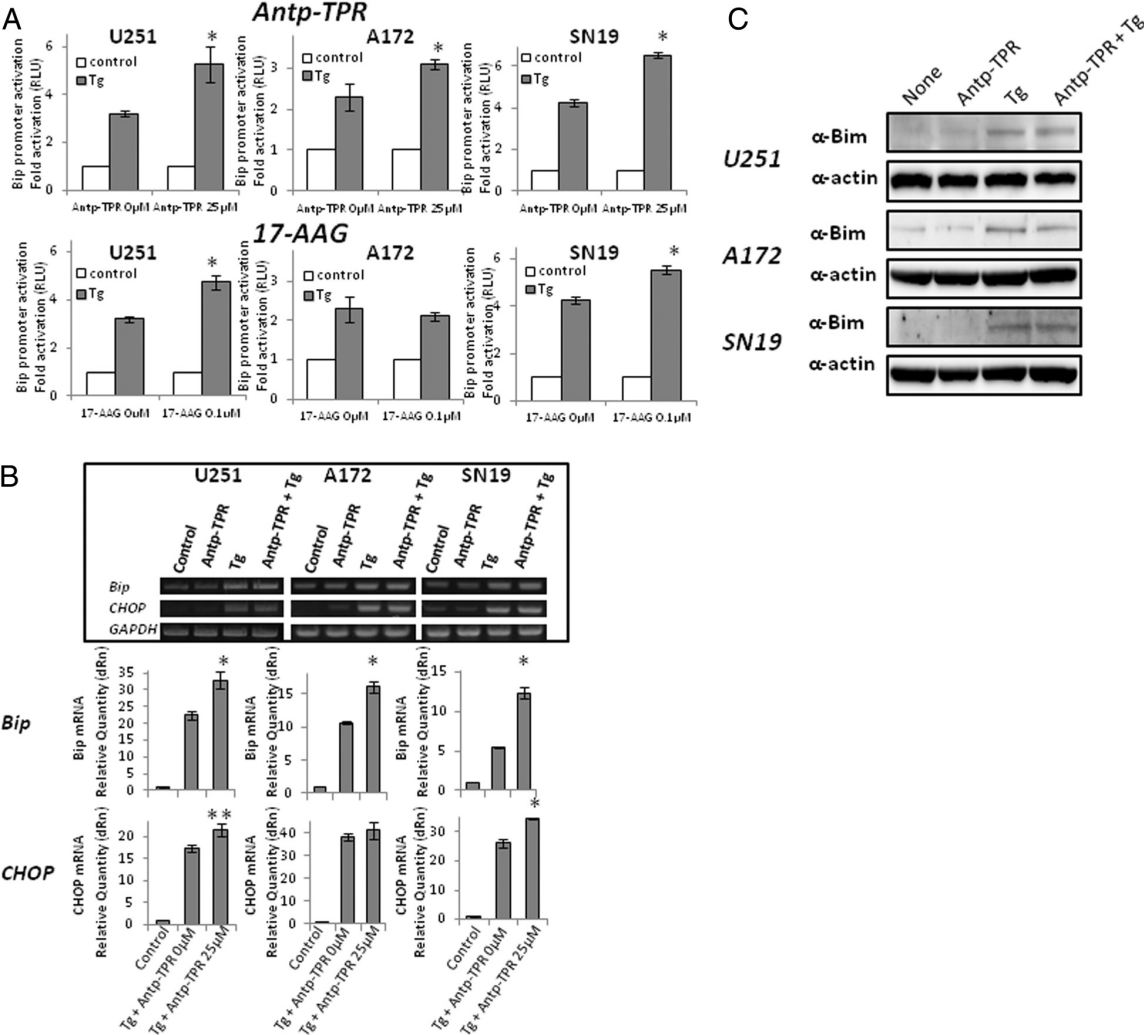
**Increase of the erUPR by Antp-TPR peptide.** (**A**) The increase of Bip promoter activation by either Antp-TPR peptide or 17-AAG. GB cells (U251, A172, and SN19) were transfected with pBipPro-Luc and exposed to ER stress. Cells were incubated with or without Antp-TPR peptide or 17-AAG for 18 h after 1 h of treatment with Tg, and then a reporter assay was performed using the Dual-Glo Luciferase assay system as described in the Materials and Methods section. Data represent the mean ± SD (*P < 0.01 compared to control cells without Antp-TPR or 17-AAG). (**B**) Effect of Antp-TPR peptide on the transcriptional level of Bip and CHOP after induction of the erUPR. Total RNA isolated from GB cells treated with or without Antp-TPR peptide after the induction of the erUPR by Tg treatment was reverse-transcribed to cDNA, and then assessed by PCR (inset) or real-time quantitative PCR using specific primers for Bip and CHOP as described in the Materials and Methods section. GAPDH served as an internal control. Histogram represent the mean ± SD (*P < 0.01 and **P < 0.05 compared to Tg + Antp-TPR 0 μM group, respectively). (**C**) Effect of Antp-TPR peptide on expression level of Bim after induction of the erUPR. GB cells were treated with or without Antp-TPR peptide after induction of the erUPR by Tg treatment, and then examined by Western-blot analysis to determine the expression level of Bim. Bands were visualized by chemiluminescence and β-actin was used as the loading control.

It was previously reported that 17-AAG induced the erUPR in rat histiocytoma [21], and in the current study we found that Antp-TPR has a different mechanism of action for cancer-cell killing compared with the conventional small-compound inhibitors targeting Hsp90 (Figure 3B and C). These evidences prompted us to investigate the action of this peptide in GB cells in relation to the erUPR. Thus, we next examined the effect of Antp-TPR on the erUPR in GB cells (U251, A172, and SN19). As shown in Figure 4A, it was found that Antp-TPR also increased the activation of Bip promoter activity in GB cells that were transiently transfected with the reporter gene plasmid pBipPro-Luc [28] after induction of the erUPR by Thapsigargin (Tg), which induces the depletion of Ca^2+^ in the ER. And a similar result was found with 17-AAG in GB cells except for A172 cells. This increase in the activation of Bip promoter activity by Antp-TPR was also found in the erUPR condition induced by tunicamycin (data not shown). In addition, Antp-TPR increased the transcriptional levels of Bip and CHOP after induction of the erUPR in GB cells (Figure 4B). On the other hand, the promoter activity of YME1L1 and GCP60, which are the respective mtUPR and Golgi apparatus stress responsive genes, as described previously [28,29], were not increased by Antp-TPR after the induction of mtUPR or Golgi stress (Additional file 4A). These results indicate that treatment with Antp-TPR peptide increases the erUPR specifically in GB cells. When we examined the effect of Antp-TPR on the expression level of Bim after induction of the erUPR in GB cells, it was found that the up-regulation of Bim after erUPR induction was not dramatically increased by treatment with Antp-TPR in this condition (Figure 4C).

### Increase of cytotoxic activity of Antp-TPR peptide in the erUPR

**Figure 5:**
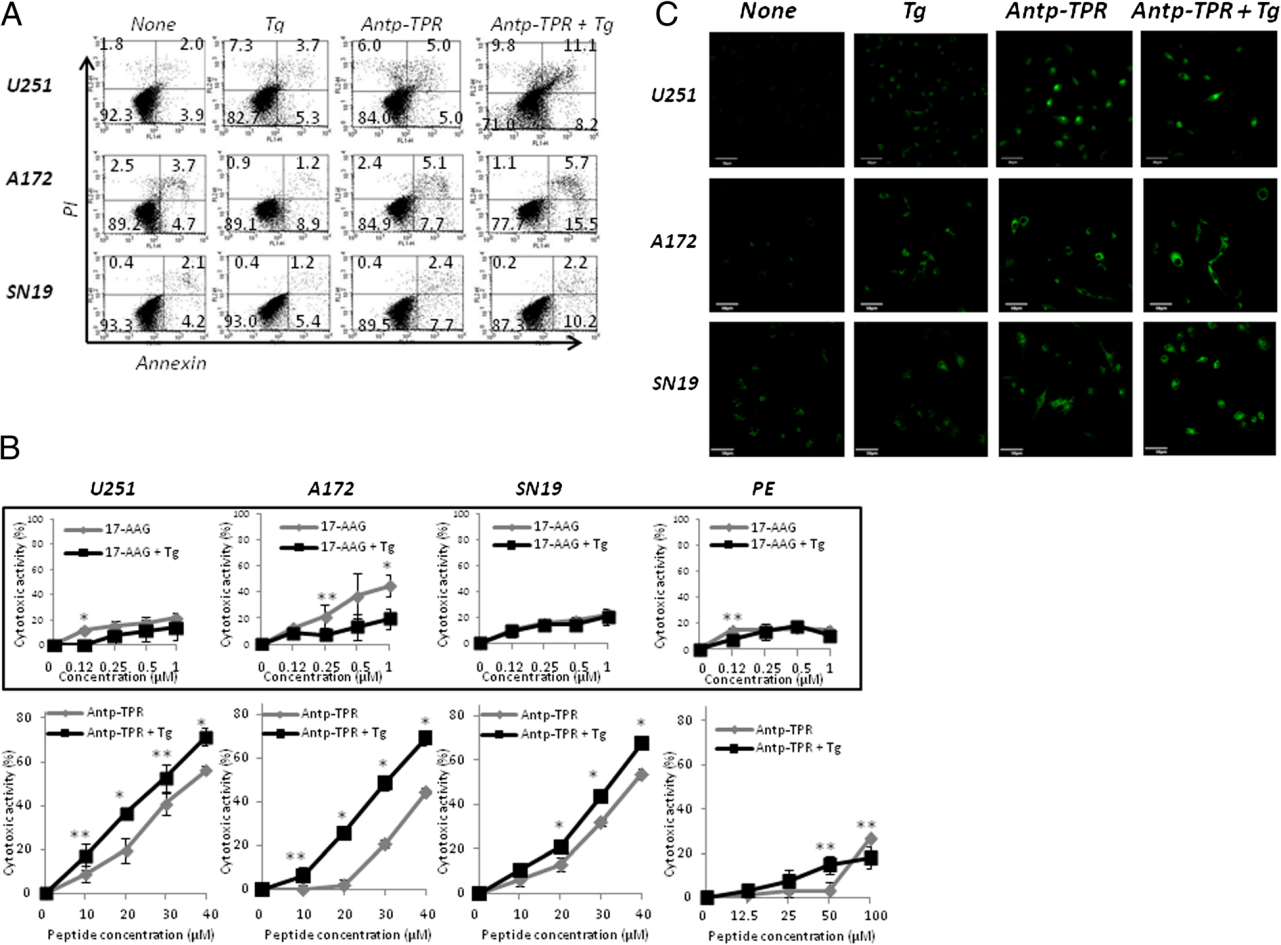
**Characterization of Antp-TPR peptide-induced GB cell killing after induction of the erUPR.** (**A**) Multiparametric flow cytometry. GB cells (U251, A172, and SN19) were incubated with or without Antp-TPR peptide for 18 h in the presence or absence of 0.1 μM Tg, and analyzed for annexin and propidium iodide (PI) staining. (**B**) The increase of cytotoxic activity of Antp-TPR peptide in the erUPR condition. GB and normal PE (ACBRI 515) cells were incubated with or without Antp-TPR peptide or 17-AAG (inset graph) at the indicated concentrations for 24 h in the presence or absence of Tg (0.05 μM), and cytotoxic activity was determined by WST-8 assay as described in the Materials and Methods section. Data represent the mean ± SD from experiments performed in triplicate. The single and double asterisks indicate *P* < 0.01 and *P* < 0.05, respectively. (**C**) Mitochondrial membrane potential. After treatment with or without Antp-TPR peptide in the presence or absence of Tg, GB cells were labeled with the mitochondrial transmembrane potential-sensitive fluorescent dye JC-1, and then images were taken using confocal laser scanning microscope as described in the Materials and Methods section. All scale bars are 50 μm.

We next characterized Antp-TPR peptide-induced cytotoxicity in GB cells after induction of the erUPR. As shown in Figure 5A, the treatment of GB cells with Antp-TPR increased the population of annexin-positive cells in the erUPR condition; however, 17-AAG did not cause such an increase in the erUPR condition (Additional file 4B). When we examined the cytotoxic activity of Antp-TPR peptide towards GB cells in the erUPR condition, interestingly, it was found that Antp-TPR caused the elevation of cytotoxic activity against GB cells tested in this study, but 17-AAG did not show an increase in cytotoxic activity toward GB cells under the conditions (Figure 5B). In addition, neither Antp-TPR nor 17-AAG increased the cytotoxic activity toward normal PE cells (ACBRI 515) in the erUPR condition with concentration-dependent manner (Figure 5B). When we examined the effect of Antp-TPR on mitochondrial membrane potential after induction of the erUPR in GB cells, it was found that treatment with Antp-TPR did not dramatically alter disruption of the mitochondrial membrane potential by the erUPR (Figure 5C), and 17-AAG also did not affect the mitochondrial membrane potential after induction of the erUPR (Additional file 4C). These results indicate that Antp-TPR peptide has selective cytotoxic activity that allows it to discriminate between cancer and normal cell lines, and that it induces an increase of this activity against cancer cells in the erUPR condition.

## Discussion

In this study we have shown that global subcellular targeting of the Hsp90 network with Antp-TPR hybrid peptide provides effective cytotoxic activity against GB cells (U251, A172, and SN19). It was found that Antp-TPR peptide mechanistically induced a simultaneous degradation of multiple Hsp90 client proteins such as p53, CDK4, Akt, and cRaf in the cytosol, triggering cancer-cell killing. GB cells are dependent on a range of activated oncoproteins and signaling pathways that require Hsp90 function [3]. Thus, Hsp90 inhibitors have been interesting agents with which to improve treatment results in GB, a primary brain tumor with a particularly dismal prognosis [30]. Although Hsp90-based therapy has been intensely pursued as a paradigm of network-oriented drug discovery [31-33], the clinical results by these agents have so far been inferior to expectations, producing only small gains in cancer patients [33]. In this study we found that the molecular activity of Antp-TPR is diverse from 17-AAG in its cancer-cell-killing mechanism (Figures 2 and 3, Additional files 2 and 3). In addition, Antp-TPR peptide did not cause the up-regulation of Hsp27, Hsp70, and Hsp90 proteins after treatment with this peptide (Figures 2 and 3). It was previously reported that the conventional Hsp90 ATPase inhibitors induce a compensatory up-regulation of Hsp70 that likely correlates with the decrease of anticancer activity [24,25]. It was also reported that 17-AAG induces the up-regulation of Hsp27, elevation of glutathione concentration, and resistance to this compound through a glutathione-mediated mechanism in cancer cells [34]. We also found that Antp-TPR peptide did not increase the concentrations of glutathione after treatment with this peptide in cancer cells (data not shown). Taking together this evidence and the results of our study, Antp-TPR hybrid peptide might have an additional advantage over Hsp90-targeted small compounds such as geldanamycin and 17-AAG.

Recently, Saito et al. [35] reported that the antidiabetic biguanides metformin, buformin, and phenformin could work as erUPR modulators during glucose deprivation in cancer cells, and that disrupting the erUPR could be an attractive approach for selective cancer-cell killing. Meanwhile, depending on the type of tumor, compounds which induce and increase the erUPR can also be used as anticancer agents such as the proteasome inhibitor bortezomib or the Hsp90 inhibitors geldanamycin and 17-AAG [36,37]. In this study, it was also found that Antp-TPR increased the erUPR through both activation of Bip promoter and up-regulation of transcriptional levels of Bip and CHOP, and then elevated the cytotoxic activity against GB cells (Figures 4 and 5). Since the Antp-TPR peptide did not increase the promoter activity of YME1L1 and GCP60 after induction of the mtUPR and Golgi stress (Additional file 4A), it is suggested that Antp-TPR might affect the erUPR specifically in cancer cells. In addition, Antp-TPR peptide did not increase cytotoxic activity against normal cells even in the erUPR condition (Figure 5). Interestingly, although 17-AAG also increased activation of the Bip promoter, the effective enhancement of the cytotoxic activity to GB cells by this compound was not observed (Figures 4 and 5). Thus, it is possible that Hsp proteins such as Hsp70 and Hsp90, up-regulated by the treatment of 17-AAG, might prevent the enhancement of cytotoxic activity against GB cells even during the erUPR.

It is well known that cancer cells are often exposed to hypoxia, nutrient starvation, oxidative stress, and other types of metabolic dysregulation that cause ER stress and activation of the erUPR. Depending on the duration and degree of ER stress, the erUPR can provide either survival signals by activating adaptive and antiapoptotic pathways or death signals by inducing a cell-death program. Sustained induction or repression of erUPR pharmacologically may thus have beneficial and therapeutic effects against cancer. In fact, glucose deprivation as well as hypoxia are common features in poorly vascularized solid tumor but are not observed in normal tissue. Interestingly, since Antp-TPR peptide increased cytotoxic activity against cancer cells after induction of the erUPR it is expected that it might exert effective antitumor activity if it penetrates a tumor. Although it is suggested that peptides are relatively easily inactivated by serum components in the human body, there are currently many candidate anticancer peptides which target intracellular molecules or organelles [38,39]. In fact, we previously reported that a 1 mg/kg dosage of Antp-TPR peptide displayed a significant antitumor activity in a xenograft model of human pancreatic cancer in mice [20]. This dosage of Antp-TPR *in vivo* is supposed to be lower than the concentrations estimated from IC_50_ values tested *in vitro* compared with small compounds such as 17-AAG. As mentioned above, Antp-TPR may have an advantage over small compounds in terms of effective antitumor activity.

## Conclusion

Our current data describe how Antp-TPR has the molecular features of a novel class of global Hsp90 inhibitor, which is capable of simultaneously disabling the multiple pools of client proteins to increase the erUPR in cancer cells. Such an approach might offer a new therapeutic approach for the management of heterogeneous and otherwise malignant human tumors, including GB. Taken together with our previous study [20], Antp-TPR hybrid peptide may provide a potent and novel type of selective anticancer therapy through its action as an erUPR modulator. Thus, the findings of this study will assist in the further elucidation of cancer treatment targeting Hsp90.

## Materials and methods

### Materials

Anti-Hsp90, anti-Hsp70, and anti-Hsp27 antibodies were purchased from Stressgen Bioreagents (Ann Arbor, MI, USA). Anti-c-Raf, anti-Akt, andCDK4 antibodies were purchased from Cell Signaling (Danvers, MA, USA). Anti-p-53 and anti-βactin antibodies, and 2-phenylethynesulfonamide (PES; also called pifithrin-μ) were purchased from Sigma (St Louis, MO, USA). 17-AAG was purchased from InvivoGen (San Diego, CA, USA). Thapsigargin (Tg), tunicamycin, and other reagents were mostly obtained from Nacalai Tesque (Kyoto, Japan). All reagents were of reagent grade.

### Cells and cell culture

Human GB cell lines (A172 and SN19) were purchased from the American Type Culture Collection (ATCC, Manassas, VA, USA), and U251 cell line was obtained from the National Cancer Institute, Frederick Cancer Research Facility, Division of Cancer Treatment Tumor Repository Program (Frederick, MD, USA). The normal pancreatic epithelial (PE) cell line ACBRI 515 was purchased from the European Collection of Cell Culture (ECACC, Salisbury, UK). Cells were cultured in RPMI-1640 (U251, A172, and SN19 cells) or CSC (ACBRI 515 cells) containing 10% fetal bovine serum, 100 μg/ml penicillin, and 100 μg/ml streptomycin at 37°C and in an atmosphere of 5% CO_2_/95% air.

### Peptide synthesis

Peptides used in this study were synthesized by the American Peptide Company (Sunnyvale, CA, USA). Peptides were dissolved in water and buffered to pH 7.4 as described previously [20]. The TPR sequence 301 K-312 K (KAYARIGNSYFK; TPR) [20] was made cell-permeable by addition of helix III of the cell-penetrating Antennapedia homeodomain sequence (underlined) [40], as follows: RQIKIWFQNRRMKWKKKAYARIGNSYFK (Antp-TPR).

### Western blotting

Western blot analyses were carried out as described previously [20,41]. Briefly, protein extracts were prepared from cells lysed with buffer containing 1% (v/v) Triton X-100, 0.1% (w/v) SDS, and 0.5% (w/v) sodium deoxycholate, separated by SDS-PAGE and transferred to nitrocellulose filters by iBlot system (Invitrogen, Carlsbad, CA, USA) according to the manufacturer’s protocol. Quenched membranes were probed with antibodies and analyzed using enhanced chemiluminescence reagent (GE Healthcare Bioscience, Uppsala, Sweden) with a LAS-3000 LuminoImage analyzer (Fujifilm, Tokyo, Japan).

### RT-PCR

The total RNA in cells was isolated using the NucleoSpin RNA kit (Macherey-Nagel, Düren, Germany). For RT reactions 0.5 μg of RNA sample was used, and the reaction was performed in a final volume of 10 μl of reaction mixture using the ReverTraAce RT kit (TOYOBO, Osaka, Japan). Each 1 μl aliquot of cDNA was amplified in a final volume of 50 μl of PCR mixture. Specific primers were as follows: cRaf, 5′-TGCAGTAAAGATCCTAAAGGTTGTC-3′ and 5′-AATTAGCTGGAACATCTGAAACTTG-3′; Akt, 5′-GACTGACACCAGGTATTTTGATGAG-3′ and 5′-ATTAAATACAGATCATGGCACGAG-3′; CDK4, 5′-GAGCTCTGCAGCACTCTTATCTACA-′ and 5′-GTCATTAAGGCAGCAAAGTAATCTCT-3′; Bip, 5′-TCTACAGCTTCTGATAATCAACCAAC-3′ and 5′-TCATTGGTGATTGTGATCTTATTTTT-3′; CHOP, 5′-ATCAAAAATCTTCACCACTCTTGAC-3′ and 5′-ACTTTCCTTTCATTCTCCTGTTCTT-3′; Hsp27, 5′-GCAGGACGAGCATGGCTACA-3′ and 5′-CTCGTTGGACTGCGTGGCTA-3′; Hsp70, 5′-GCCATGACGAAAGACAACAAT-3′ and 5′-CTTTGTACTTCTCCGCCTCCT-3′; Hsp90, 5′-ACTACACATCTGCCTCTGGTGATGA-3′ and 5′-TGTTTCCGAAGACGTTCCACAA-3′; glyceraldehyde-3-phosphate dehydrogenase (GAPDH), 5′-GTCTTCACCACCATGGAGAAGGCT-3′ and 5′-CATGCCAGTGAGCTTCCCGTTCA-3′. GAPDH was used as an internal control.

PCR product was run on a 1% agarose gel for UV analysis.

### Quantitative real-time PCR analysis

Quantitative real-time PCR analysis was carried out using the SYBR Green Real-time PCR Master Mix kit (TOYOBO) on the Mx3000p Real-time QPCR System (Stratagene, La Jolla, CA, USA). Amplification was performed under the following conditions: after an initial denaturation step of 95°C for 60 s: 45 cycles of 95°C for 15 s, 60°C for 15 s, and 72°C for 45 s. The primers were the same as for RT-PCR analysis (see previous section).

### Assay for cell viability

Cell viability was determined by WST-8 assay as described previously [20]. Briefly, cells were seeded onto 96-well plates at 2000–3000 cells/well. After incubating with the test peptides, the assay for cell viability was carried out using Living Cell Count Reagent SF (Nacalai Tesque) according to the manufacturer’s protocol. Absorbance was measured at a wavelength of 450 nm using a 96-well microplate reader (GE Healthcare Bioscience). Cytotoxic activity was calculated from the percentage of cell viability; 0% cell viability was defined as 100% cytotoxic activity (100% cell-killing activity).

### Reporter assay

A reporter assay was carried out as described previously [35]. Briefly, GB cells (U251, A172, and SN19) were transfected with firefly luciferase-containing reporter plasmids of the Bip promoter (pBipPro-Luc), in which the Bip promoter region was cloned as described previously [28]. The *Renilla* luciferase-containing plasmid pRL-SV40 (Promega, Madison, WI, USA) was used as an internal control. The relative activity of firefly luciferase to *Renilla* luciferase (mean ± SD from triplicate determinations) was determined using the Dual-Glo Luciferase Assay System (Promega). 0.05-0.5 μM of Tg was used for the induction of erUPR depend on the number of cells. Induction of mitochondrial UPR (mtUPR) and Golgi apparatus stress was carried out as described previously [28,29].

### Flow cytometry assay

Flow cytometry assay was performed as described previously [20,42]. Briefly, after incubation with or without Antp-TPR peptide or Tg, cells were collected and washed twice with PBS. Following this, the cell pellets were resuspended. Flow cytometry (Becton Dickinson) analysis was performed using the Annexin V-Fluorescein Staining Kit (Wako) according to the manufacturer’s protocol. Data were analyzed using CellQuest Software.

### Analysis of mitochondrial membrane potential

Change of mitochondrial membrane potential was evaluated as described previously [43]. Briefly, cells were labeled for 30 min with 5 μg/ml mitochondrial membrane potential-sensitive fluorescent dye, JC-1 (Invitrogen, Carlsbad, CA, USA), in a glass-bottomed dish after treatment with or without Antp-TPR or Tg, and then confocal images were taken using an Olympus FV 1000 confocal laser scanning microscope (Olympus, Tokyo, Japan).

### Statistical analysis

All values are expressed as the mean ± SD and statistical significance was determined using the Student *t*-test with statistical significance assessed with a probability value less than 0.05.

## Abbreviations

Antp: antennapedia homeodomain sequence; ATF6: activation transcription factor 6; GB: glioblastoma; CDK: cyclin dependent kinase; EGF: epidermal growth factor; Her2: human epidermal growth factor receptor type 2; Hop: p60/Hsp-organizing protein; Hsp90: heat shock protein 90; IC50: the peptide concentration inducing 50% inhibition of cell growth; PES: 2-phenylethynesulfonamide; PI: propidium iodid; PTEN: phosphate and tensin homolog deleted on chromosome 10; Raf: proto-oncogene serine/threonine-protein kinase; Tg: thapsigargin; TPR: tetratricopeptide repeat.

## Competing interests

The authors declare that they have no competing interests.

## Authors’ contributions

TH, MK, and KK designed this research work. TH, AT, and MK performed experiments and analyzed data in this manuscript. TH, MK, and KK, interpreted the data and wrote the manuscript. All authors read and approved the final manuscript.

## Supplementary Material

Additional file 1 **Analysis of PTEN expression in human breast (BT20 and T47D), lung (H460, A549, H322, and H1299), colon (COLO320HSR), and prostate (DU145) cancer, epithelial carcinoma (A431), and GB (U251) cell lines.** Cell extracts from the indicated cell lines were examined for PTEN expression by Western-blot analysis with antibodies against PTEN. β-Actin was used as the loading control. Bands were visualized by chemiluminescence as described in the Materials and Methods section. Click here for file

Additional file 2 **Effect of 17-AAG on Hsp90, Hsp70, Hsp27, and Akt expression in GB cells.** A172 cells were treated with different concentrations of 17-AAG for 24 h. After 24 h cells were harvested and examined for Hsp90, Hsp70, Hsp27, and Akt expression by Western-blot analysis with antibodies against these proteins. β-Actin was used as the loading control. Click here for file

Additional file 3 **Time course of cell viability after treatment with Antp-TPR or 17-AAG (A) and effect of Hsp70 inhibitor on the cytotoxic activity of Antp-TPR or 17-AAG to U251 cells (B).** (A) U251 cells were treated with Antp-TPR or 17-AAG at the indicated concentrations, and then analyzed for cell viability by WST-8 assay. (B) U251 cells were treated with Antp-TPR or 17-AAG at the indicated concentrations in the presence or absence of PES (2.5 μM), and then cell viability was assessed by WST-8 assay. Data represent the mean ± SD. Click here for file

Additional file 4 **Effect of Antp-TPR peptide on up-regulation of the YME1L1 and GCP60 genes under the mtUPR or Golgi stress condition (A) and Characterization of 17-AAG-induced GB cell killing after induction of the erUPR (B and C).** (A) U251 cells were transfected with YME1L1 and GCP promoter-reporter constructs and exposed to mitochondrial or Golgi stress as described in the Materials and Methods section. After induction of mtUPR or Golgi stress, cells were incubated with or without Antp-TPR peptide for 18 h, and then reporter assay was performed by the Dual-Glo Luciferase assay system. Data represent the mean ± SD. (B) Multiparametric flow cytometry. U251 cells were incubated with or without 17-AAG peptide for 18 h in the presence or absence of 0.5 μM Tg, and analyzed for annexin and propidium iodide (PI) staining. (C) Mitochondrial membrane potential after treatment with 17-AAG. After treatment with or without 17-AAG in the presence or absence of Tg, U251 cells were labeled with JC-1 and then images were taken using a confocal laser scanning microscope as described in the Materials and Methods section. All scale bars are 50 μm. Click here for file
