# Supplementary figures and images for: Molecular mechanism of cytotoxicity induced by Hsp90-targeted Antp-TPR hybrid peptide in glioblastoma cells
Source: Mol Cancer. 2012 Aug 22;11:59. doi: 10.1186/1476-4598-11-59 (PMC3499401; doi:10.1186/1476-4598-11-59)

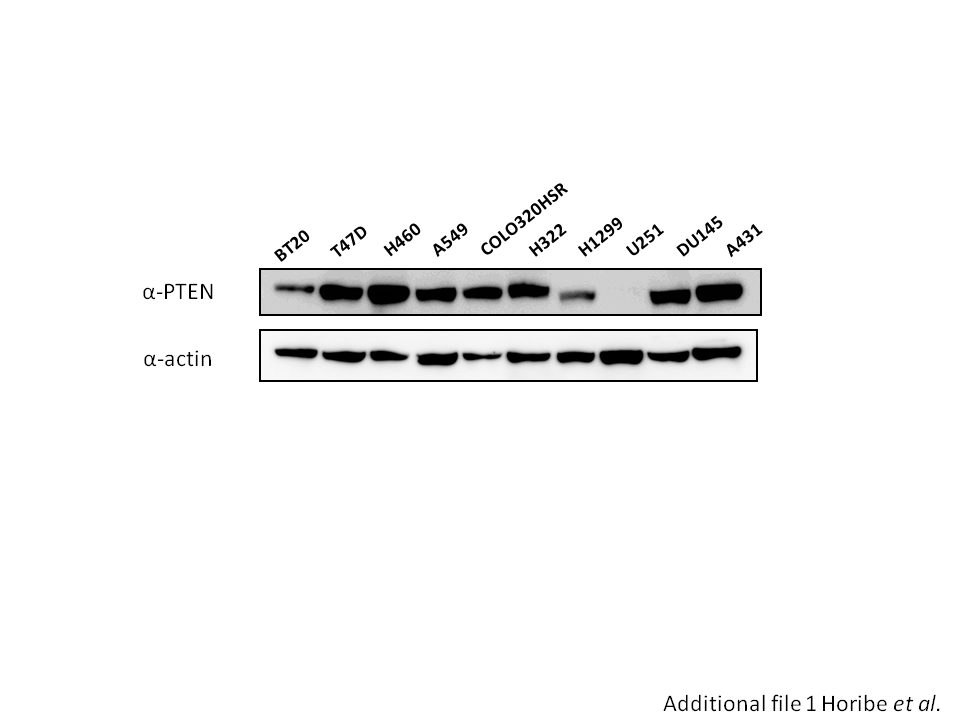

Supplement: Additional file 1 — Analysis of PTEN expression in human breast (BT20 and T47D), lung (H460, A549, H322, and H1299), colon (COLO320HSR), and prostate (DU145) cancer, epithelial carcinoma (A431), and GB (U251) cell lines. Cell extracts from the indicated cell lines were examined for PTEN expression by Western-blot analysis with antibodies against PTEN. β-Actin was used as the loading control. Bands were visualized by chemiluminescence as described in the Materials and Methods section. [file 1476-4598-11-59-S1.tiff]

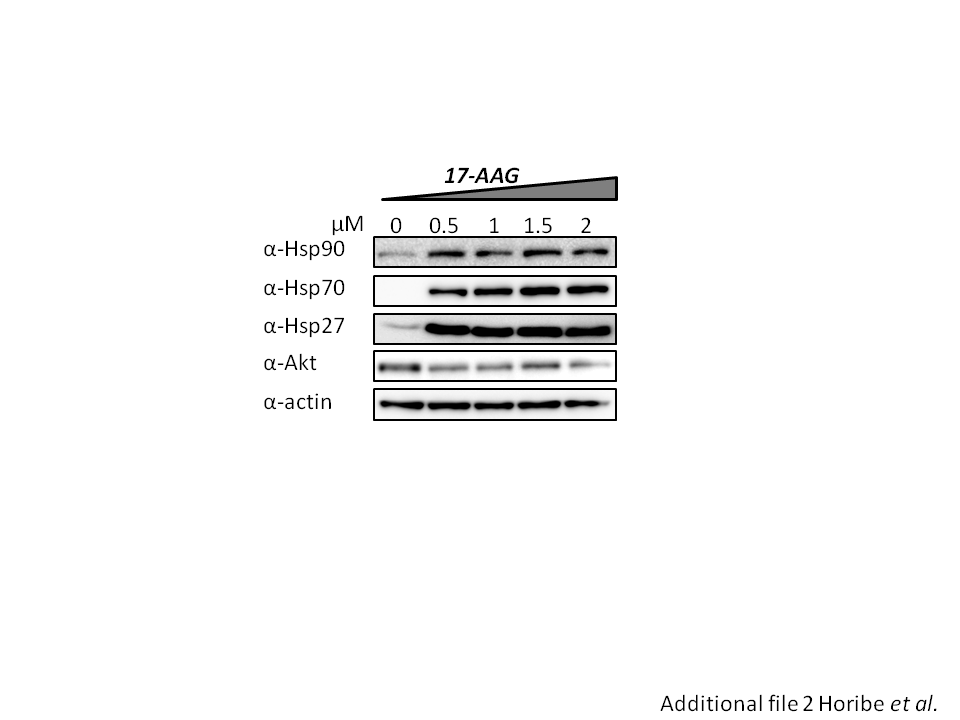

Supplement: Additional file 2 — Effect of 17-AAG on Hsp90, Hsp70, Hsp27, and Akt expression in GB cells. A172 cells were treated with different concentrations of 17-AAG for 24 h. After 24 h cells were harvested and examined for Hsp90, Hsp70, Hsp27, and Akt expression by Western-blot analysis with antibodies against these proteins. β-Actin was used as the loading control. [file 1476-4598-11-59-S2.tiff]

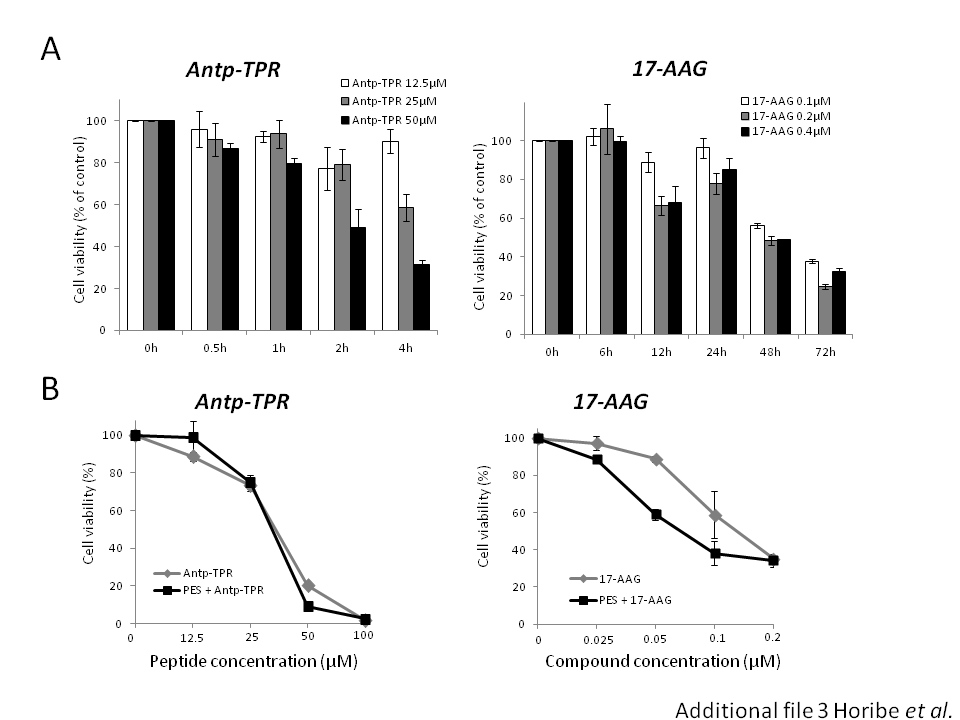

Supplement: Additional file 3 — Time course of cell viability after treatment with Antp-TPR or 17-AAG (A) and effect of Hsp70 inhibitor on the cytotoxic activity of Antp-TPR or 17-AAG to U251 cells (B). (A) U251 cells were treated with Antp-TPR or 17-AAG at the indicated concentrations, and then analyzed for cell viability by WST-8 assay. (B) U251 cells were treated with Antp-TPR or 17-AAG at the indicated concentrations in the presence or absence of PES (2.5 μM), and then cell viability was assessed by WST-8 assay. Data represent the mean ± SD. [file 1476-4598-11-59-S3.tiff]

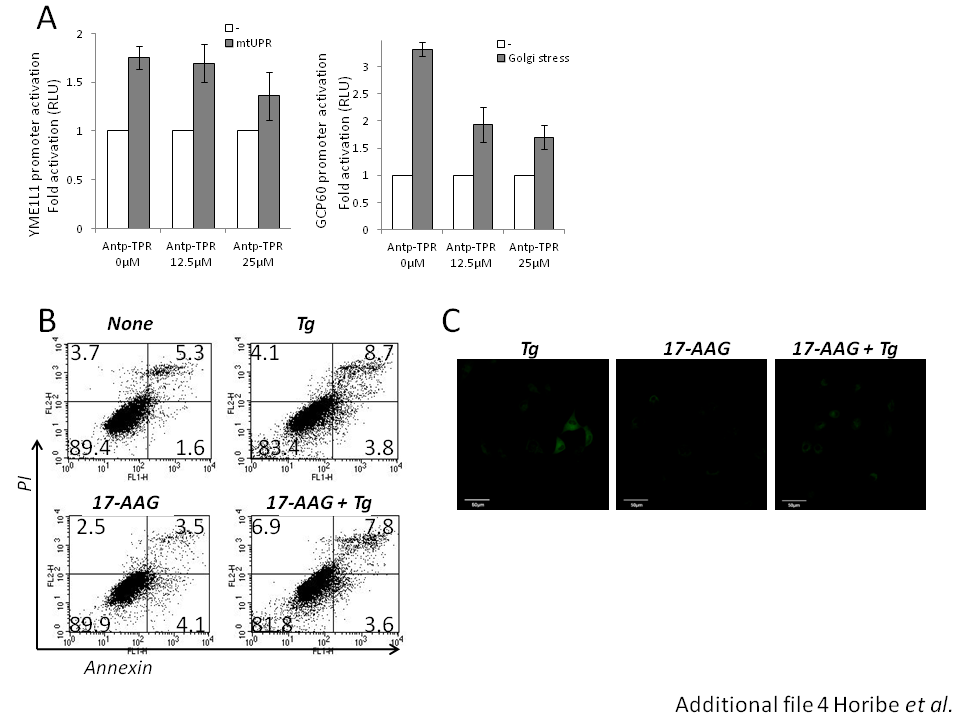

Supplement: Additional file 4 — Effect of Antp-TPR peptide on up-regulation of the YME1L1 and GCP60 genes under the mtUPR or Golgi stress condition (A) and Characterization of 17-AAG-induced GB cell killing after induction of the erUPR (B and C). (A) U251 cells were transfected with YME1L1 and GCP promoter-reporter constructs and exposed to mitochondrial or Golgi stress as described in the Materials and Methods section. After induction of mtUPR or Golgi stress, cells were incubated with or without Antp-TPR peptide for 18 h, and then reporter assay was performed by the Dual-Glo Luciferase assay system. Data represent the mean ± SD. (B) Multiparametric flow cytometry. U251 cells were incubated with or without 17-AAG peptide for 18 h in the presence or absence of 0.5 μM Tg, and analyzed for annexin and propidium iodide (PI) staining. (C) Mitochondrial membrane potential after treatment with 17-AAG. After treatment with or without 17-AAG in the presence or absence of Tg, U251 cells were labeled with JC-1 and then images were taken using a confocal laser scanning microscope as described in the Materials and Methods section. All scale bars are 50 μm. [file 1476-4598-11-59-S4.tiff]
